# Simultaneous efficacy of IL-15 and the HSP70 fragments in increasing immunostimulatory effects of a HIV-1 DNA vaccine candidate against SCR HIV-1

**DOI:** 10.1371/journal.pone.0336042

**Published:** 2025-11-06

**Authors:** Mehrshad Fekri, Azam Bolhassani, Monireh Movahedi, Fereshteh Rahmati

**Affiliations:** 1 Department of Biochemistry, NT.C., Islamic Azad University, Tehran, Iran; 2 Department of Hepatitis, AIDS and Blood-borne Diseases, Pasteur Institute of Iran, Tehran, Iran; Henan University, CHINA

## Abstract

Interleukin (IL)-15 serves as a potent adjuvant that can enhance T cell-directed vaccine responses. Moreover, heat shock proteins (HSPs) have been used as potent adjuvants in immunotherapy of tumors and infectious diseases, as well as in vaccine development. In this study, we evaluated the ability of IL-15 and HSP70 fragments (C-terminal (CT)-Hsp70 and N-terminal (NT)-Hsp70) to act as adjuvants for enhancing the immunostimulatory effects of a HIV-1 DNA vaccine candidate against single-cycle replicable (SCR) HIV-1. These adjuvants were administered individually and in combination with the HIV-1 Nef antigen candidate in BALB/c mice. The Nef, CT-Hsp70-Nef, NT-Hsp70-Nef and IL-15 genes were individually subcloned into the eukaryotic expression vector pVAX-1. Our results showed that the linkage of the CT-Hsp70 or the NT-Hsp70 gene to the Nef gene could significantly increase immune responses compared to the Nef gene alone (*p* < 0.05). Moreover, the combination of pVAX-IL-15 with DNA constructs could significantly augment immune responses. Indeed, the combination of pVAX-IL-15 with pVAX-CT-Hsp70-Nef or pVAX-NT-Hsp70-Nef could substantially increase immune responses compared to pVAX-Nef combined with pVAX-IL-15 (*p* < 0.05). Notably, the pVAX-CT-Hsp70-Nef construct, alone or combined with pVAX-IL-15, could significantly enhance immune responses to a greater extent than pVAX-NT-Hsp70-Nef with or without pVAX-IL-15 (*p* < 0.05). In addition, we demonstrated that the highest secretion of total IgG antibody, interferon (IFN)-**γ**, and granzyme B was observed in the group receiving pVAX-CT-Hsp70-Nef combined with pVAX-IL-15, suggesting a shift in immune responses toward T helper type 1 (Th1) and cytotoxic T lymphocyte (CTL) activities. Furthermore, the pVAX-CT-Hsp70-Nef combined with pVAX-IL-15 regimen could maintain IFN-γ secretion after infection of mouse splenocytes with SCR HIV-1. Overall, our findings indicated that the concurrent use of two adjuvants—CT-Hsp70 and IL-15—effectively enhances antigen-specific immune responses. This regimen could be utilized as a vaccine candidate for boosting effective immune responses against HIV-1 infection.

## Introduction

Interleukin (IL)-15 is a crucial pro-inflammatory cytokine composed of two subunits, known for its role in supporting long-term immune surveillance via enhancement of cytotoxic lymphocyte function [1]. This cytokine directly induces production of interferon (IFN)-γ by lymphocytes, working synergistically with type I and II IFNs to establish an early innate immune defense against diverse pathogens. In addition to its direct effects, IL-15 indirectly modulates the functionality of antigen-presenting cells (APCs) such as macrophages and dendritic cells (DCs), stimulating the secretion of interferon-induced chemokines like IP-10/CXCL10 in an IFN-γ-dependent manner [2–5].

Functioning as a potent immunoregulatory molecule, IL-15 activates human DCs and natural killer (NK) cells, supports memory T cell homeostasis, enhances effector T cell responses, and provides anti-apoptotic signals to prolong T cell viability [6,7]. Its unique ability to potentiate T cell responses has prompted its development as an immunological adjuvant, particularly for vaccines aimed at eliciting Th1-mediated immunity [8]. Preclinical models have further shown that IL-15 can break immune tolerance and stimulate effective anti-tumor mechanisms [9]. DCs matured in the presence of IL-15 are being investigated as promising components of next-generation DC-based immunotherapies; however, these approaches may require combinatorial strategies to achieve clinically relevant immunity, given the complexity of tumor immune evasion [10]. Clinical studies have explored the synergistic impact of IL-7 and IL-15 when used as vaccine adjuvants [11–14], with additional applications in oncological immunotherapy [15] and in reversing T cell dysfunction during chronic human immunodeficiency virus (HIV)-1 infection [16].

In parallel, heat shock proteins (HSPs), endogenous molecular chaperones involved in protein folding and stress response, have gained attention as natural adjuvants in vaccine development. Through their interaction with surface receptors on APCs, HSPs enhance antigen cross-presentation, positioning them as powerful carriers for covalently linked antigens [17]. These proteins have been harnessed in both infectious disease and cancer vaccines due to their robust immunostimulatory capacity. In particular, terminal fragments of HSPs—known as mini-chaperones—retain their peptide-binding ability and efficiently target antigens to APCs via CD91 and scavenger receptor class A leading to improved cytotoxic T lymphocyte (CTL) activation in infectious disease models [18]. Among the HSP family members, Hsp70, gp96, and Hsp90-based vaccines have been extensively studied, with promising outcomes reported across various animal models and clinical trials involving cancer immunotherapy [19].

Motivated by these findings on IL-15 and HSPs as powerful immune enhancers, the present study investigates a DNA-based vaccination strategy combining these two adjuvants. Specifically, we evaluated plasmid constructs encoding HSP70 fragments (either C-terminal or N-terminal domains) genetically fused with the HIV-1 negative regulatory factor (Nef) antigen, either alone or in combination with an IL-15-encoding plasmid, to determine their capacity to elicit Nef-specific cellular immunity. Nef, a HIV accessory protein, is expressed early in the infectious cycle, and introduced as a potent immunogen in eliciting humoral and cellular immunity [20]. DNA vaccines are well-recognized for inducing targeted immune responses, and recent efforts continue to focus on improving their immunogenicity, especially for pathogens like HIV-1. Here, we conducted a preclinical assessment of the synergistic effects of IL-15 and HSP70 constructs in strengthening the immunostimulatory profile of a candidate DNA vaccine targeting single-cycle replicable (SCR) HIV-1.

## Materials and methods

### Construction of four recombinant eukaryotic vectors

We constructed the pVAX-NT-Hsp70-Nef and pVAX-CT-Hsp70-Nef plasmids by isolating the NT-Hsp70-Nef and CT-Hsp70-Nef gene inserts from our previously established bacterial vectors, pET23a-NT-Hsp70-Nef and pET23a-CT-Hsp70-Nef [21], and subcloning them into the *Bam*HI/*Xho*I sites of the mammalian expression vector pVAX-1. Similarly, to develop pVAX-IL-15, we excised the IL-15 gene from our validated pcDNA-IL-15 plasmid using *Nhe*I and *Pst*I enzymes and ligated it into the corresponding sites of pVAX-1. The pVAX-Nef construct was previously produced in our laboratory by insertion of the Nef gene into the *Nhe*I and *Bam*HI restriction sites of pVAX-1. All recombinant DNA plasmids were purified using the EndoFree Plasmid Giga Kit (Qiagen) to ensure low endotoxin content, suitable for *in vivo* experiments. DNA concentration and purity were determined using a NanoDrop spectrophotometer.

### Generation of pET24a-IL-15 and expression of the recombinant IL-15 protein

To construct the prokaryotic IL-15 expression vector, the IL-15 gene was amplified from our existing pcDNA-IL-15 plasmid, and cloned into the pET24a vector at the *Nhe*I/*Xho*I restriction sites. For the recombinant protein production, transformation was carried out in both *E. coli* Rosetta and BL21 strains, followed by induction using 1 mM IPTG when cultures reached an optical density (OD_600_) of 0.6–0.7. Protein expression was monitored at multiple time points (2, 4, and 16 hours post-induction) using SDS-PAGE, and identity was confirmed by western blot analysis employing a horseradish peroxidase (HRP)-conjugated anti-His tag antibody (1:10,000 *v/v* dilution). Protein solubility was examined according to the manufacturer’s instructions (Qiagen), and soluble fractions were selected for purification. IL-15 was isolated under native conditions using Ni-NTA agarose resin, with elution performed at pH 8.0 using 250 mM imidazole. Following purification, the protein underwent dialysis against PBS1X to remove excess imidazole buffer for downstream applications. Quantification of the purified protein was conducted using the Bradford protein assay in parallel with absorbance-based measurement via NanoDrop 2000 spectrophotometry.

### Expression and purification of recombinant Nef, NT-Hsp70-Nef, and CT-Hsp70-Nef proteins

Our team had previously designed, and validated the plasmid constructs pET23a-NT-Hsp70-Nef, pET23a-CT-Hsp70-Nef [20], and pET23a-Nef [22]. The expression of recombinant NT-Hsp70-Nef, CT-Hsp70-Nef, and Nef proteins had also been established in earlier experiments [21,22]. In this study, we scaled up the production process to obtain higher yields of these target proteins for immunological evaluations.

Recombinant NT-Hsp70-Nef and CT-Hsp70-Nef proteins were produced in *E. coli* Rosetta cells, using 1 mM IPTG for induction at 37 °C. Induction periods were optimized based on protein type: 4 hours for CT-Hsp70-Nef and 16 hours for NT-Hsp70-Nef. Protein extraction and purification were performed under denaturing conditions using an 8 M urea lysis buffer (pH 4.5). The purified proteins were visualized by SDS-PAGE, showing distinct molecular weight bands of approximately ~42 kDa for CT-Hsp70-Nef and ~70 kDa for NT-Hsp70-Nef. The Nef protein was previously cloned into the pET23a vector, overexpressed in *E. coli* BL21 cells, and purified under denaturing conditions via Ni-NTA affinity chromatography. Protein expression was induced at 37 °C with 1 mM IPTG for 16–18 hours. SDS-PAGE confirmed successful isolation of the Nef protein, appearing as a distinct band of ~27 kDa.

### Mice immunization protocol

All animal-related procedures were conducted in accordance with protocols approved by the Ethics Committee of Islamic Azad University (Ethics Code: IR.IAU.TNB.REC.1404.118). Ethical guidelines were followed as the national ethical committee and in accordance with ARRIVE guidelines. Female BALB/c mice, aged 5–7 weeks, were obtained from the Pasteur Institute of Iran (Karaj branch), acclimated for 5–7 days before the experimental trials, and housed in pathogen-free conditions with controlled temperature (22 ± 2°C), humidity (50–60%), and a 12-hour light/dark cycle, and provided with a standard pellet diet and clean water ad libitum. Since the study involves the use of mice, the humane endpoints were kept under consideration throughout the study. Animals were monitored twice daily for signs of distress or illness including weight loss (>15–20%), lethargy, ruffled fur, hunched posture, reduced mobility, and abnormal respiration. For predefined humane endpoints, animals were anesthetized (*i.e.,* isoflurane-induced anesthesia) and then euthanized if they exhibited severe distress, > 20% body weight loss, paralysis or prostration, or moribund condition. For euthanasia method, cervical dislocation was performed consistent with AVMA Guidelines for the Euthanasia of Animals (2020). Survival was monitored daily until humane endpoints were reached. No procedures involving unrelieved pain or distress were performed. All efforts were made to minimize animal suffering, including the use of analgesics where applicable. No unexpected deaths occurred during the study. Moreover, while designing the study, the minimal statistical limit was kept into consideration and the least number of mice were taken for the assays. The mice were randomly assigned to nine experimental groups (n = 5 per group in each cage) and were immunized subcutaneously with designated DNA vaccine formulations. Each mouse received 50 µg of plasmid DNA per dose, administered on three occasions at two-week intervals. The specific DNA constructs delivered to each group are summarized in Table 1.

**Table 1:** Mice immunization program.

Group	Priming (Day 0)/ Booster 1 (Day 14)/ Booster 2 (Day 28)	Immunity tests (Day 50)
**G1**	Nef DNA	Antibody secretion, Cytokine secretion, Granzyme B secretion, and SCR HIV-1-specific cytokine secretion
**G2**	NT-Hsp70-Nef DNA	
**G3**	CT-Hsp70-Nef DNA	
**G4**	Nef DNA + IL-15 DNA	
**G5**	NT-Hsp70-Nef DNA + IL-15 DNA	
**G6**	CT-Hsp70-Nef DNA + IL-15 DNA	
**G7**	IL-15 DNA	
**G8**	pVAX	
**G9**	PBS	

### Assessment of antibody responses

Serum samples were collected from all mice three weeks following the final immunization to analyze humoral immune responses elicited by the DNA-based formulations. Total IgG concentrations were assessed using an indirect ELISA. Mouse sera were diluted 1:100 (*v/v*), and 96-well plates were pre-coated with recombinant proteins (Nef, IL-15, NT-Hsp70-Nef, or CT-Hsp70-Nef) at a concentration of 5 µg/mL. Detection was performed using an HRP-conjugated goat anti-mouse IgG antibody (diluted 1:10,000 *v/v*, Sigma-Aldrich), and colorimetric development was initiated using tetramethylbenzidine (TMB) substrate. The reaction was stopped, and absorbance was measured at 450 nm using a microplate reader. All experimental values were presented as the mean ± standard deviation (SD) for each group.

### Evaluation of cytokine production

Three weeks after the final DNA immunization, splenocytes were isolated from five mice per group using ACK lysis buffer to remove red blood cells, based on previously described procedures [22]. A total of 2.0 × 10^6^ viable splenocytes per mL were seeded into 24-well culture plates and incubated with 5 µg/mL of recombinant Nef, IL-15, NT-Hsp70-Nef, or CT-Hsp70-Nef proteins for 72 hours under standard culture conditions. Furthermore, Concanavalin A (Con A; 5 µg/mL) was employed as a positive control, while cells cultured with 10% RPMI-1640 medium alone served as a negative control. The concentrations of interferon-gamma (IFN-γ) and interleukin-5 (IL-5) in culture supernatants were quantified using commercial sandwich ELISA kits [Mabtech; mouse IL-5 (HRP): 3391-1H-6/3391-1H-20; mouse IFN-gamma: 3321-1H-6] following the manufacturer’s guidelines. Cytokine levels were reported as mean values ± standard deviation (SD) for each experimental group.

### Measurement of granzyme B release

To evaluate cytotoxic activity, the secretion of granzyme B by effector splenocytes (E) was assessed using a co-culture assay with SP2/0 target cells (T). Briefly, SP2/0 cells were seeded at a density of 2.0 × 10^4^ cells per well in 96-well plates (triplicate wells per condition), and incubated for 24 hours in the presence of 5 µg/mL of recombinant proteins (Nef, IL-15, NT-Hsp70-Nef, or CT-Hsp70-Nef). Following target sensitization, splenocytes isolated from immunized mice were added at an effector-to-target (E: T) ratio of 100:1, using RPMI-1640 medium supplemented with 10% fetal bovine serum (Gibco). After a 6-hour incubation period, the culture supernatants were collected and analyzed for granzyme B content using a commercial ELISA kit (BMS6029), adhering to the manufacturer’s protocol. Data are presented as mean ± standard deviation (SD) for each test group.

### *In vitro* assessment of SCR HIV-1-specific cytokine production

The generation of single-cycle replicable HIV-1 (SCR HIV-1) was carried out following previously established methods [21,22]. Briefly, HEK-293T cells (provided from the Cell bank of Pasteur Institute of Iran) were co-transfected with three plasmids—pmzNL4.3 (non-infective HIV-1 strain (mzNL4–3), containing the mutated genome; 2 μg [23]), psPAX2 (packaging plasmid, #12260l; 1.5 μg), and pMD2.G (envelope plasmid, # 12259; 0.5 μg)—using TurboFect transfection reagent (Fermentas) following the manufacturer’s protocol. Culture supernatants containing viral particles were harvested and concentrated by ultracentrifugation at 45,000 × *g* for 2 hours. The viral load (SCR HIV-1 titer) was quantified using a p24 antigen ELISA kit (RETRO-TEK; An enzyme-linked immunoassay used to detect HIV-1 p24 antigen in research samples, including cell culture medium, human sera and plasma). For cytokine stimulation assays, splenocytes (2.0 × 10^6^ cells/well) isolated from immunized mice were incubated with SCR HIV-1 corresponding to 50 µg of p24 protein for 72 hours. After incubation, the supernatants were collected and analyzed for levels of IFN-γ and IL-5 using a sandwich ELISA kit, according to the manufacturer’s instructions.

### Statistical analysis

Statistical comparisons between experimental and control groups were performed using Prism 8 software (GraphPad Software, San Diego, CA, USA) via one-way ANOVA. Data were expressed as means ± standard deviation (SD). Significance thresholds were defined as follows: *p < 0.05, **p < 0.01, ***p < 0.001, ****p < 0.0001; non-significant (ns) outcomes were assigned for *p* > 0.05.

## Results

### Verification of recombinant eukaryotic plasmids

**Fig 1:**
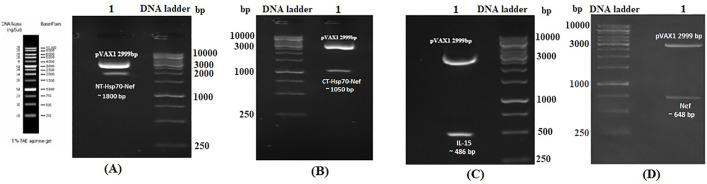
Validation of recombinant plasmid constructs via restriction digestion. **A)** Digestion of pVAX-NT-Hsp70-Nef with *Bam*HI and *Xho*I; B) pVAX-CT-Hsp70-Nef digested with the same enzyme set; **C)** Verification of pVAX-IL-15 through *Nhe*I and *Pst*I digestion; **D)** Digestion of pVAX-Nef using *Nhe*I and *Bam*HI. A 1 kb DNA ladder (Fermentas) was used as a molecular size marker.

The integrity of the recombinant plasmids—pVAX-NT-Hsp70-Nef, pVAX-CT-Hsp70-Nef, pVAX-IL-15, and pVAX-Nef—was validated through restriction enzyme digestion. As expected, digestion of pVAX-NT-Hsp70-Nef released a fragment of approximately 1800 base pairs (bp) (Fig 1A), while pVAX-CT-Hsp70-Nef produced a 1050 bp fragment (Fig 1B). Similarly, pVAX-IL-15 and pVAX-Nef digestion analyses yielded bands of 486 bp and 648 bp, respectively (Fig 1C and 1D). Plasmid DNA concentrations ranged between 2.1 and 3.4 mg/mL, and the purity ratios (A_260_/A_280_) for all preparations were within the acceptable range of 1.83 to 1.91, indicating high-quality DNA suitable for downstream experiments.

### Validation of pET24a-IL-15 and the IL-15 protein expression

**Fig 2:**
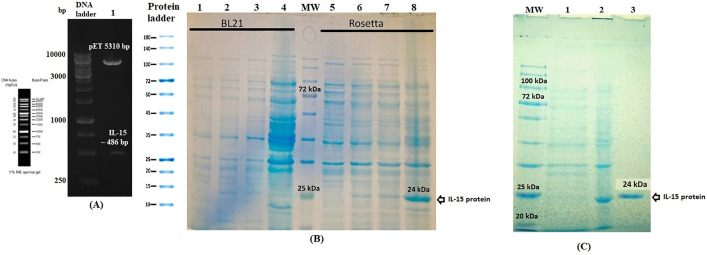
A) Restriction analysis of pET24a-IL-15 using *Nhe*I and *Xho*I; 1 kb DNA ladder (Fermentas) was shown. **B)** SDS-PAGE showing expression of IL-15 in *E. coli* strains Rosetta and BL21: Lanes 1 & 5: un-induced; Lanes 2 & 6: 2 h post-IPTG induction; Lanes 3 & 7: 4 h post-induction; Lanes 4 & 8: 16 h post-induction. MW: Protein ladder (10–180 kDa, Fermentas). **C)** SDS-PAGE analysis of purified IL-15: Lane 1: pre-induction; Lane 2: 16 h post-induction; Lane 3: Ni-NTA-purified IL-15 protein. MW marker: 10–180 kDa.

The construction of the pET24a-IL-15 expression plasmid was confirmed by enzymatic digestion, which produced a clear band of 486 base pairs corresponding to the inserted IL-15 gene segment (Fig 2A). Recombinant IL-15 protein was then expressed in *E. coli* Rosetta strain following IPTG induction at 37 °C for 16–18 hours. SDS-PAGE analysis revealed a dominant band at approximately 24 kDa, aligning with the expected size of IL-15, and this was subsequently verified via western blot using an anti-His tag antibody. The target protein was localized in the soluble fraction, allowing purification to be performed under native conditions using Ni-NTA affinity chromatography (Fig 2B and 2C). The final protein yield was 0.396 mg/mL, obtained from a 200 mL bacterial culture.

### Production of recombinant NT-Hsp70-Nef, CT-Hsp70-Nef and Nef proteins

The recombinant NT-Hsp70-Nef, CT-Hsp70-Nef and Nef proteins were generated and purified based on methodologies described in our previous publications [21,22]. Protein expression was confirmed by SDS-PAGE, which showed distinct bands corresponding to the expected molecular weights: ~ 70 kDa for NT-Hsp70-Nef, ~ 42 kDa for CT-Hsp70-Nef, and ~27 kDa for the Nef protein.

### Antibody assessment

**Fig 3:**
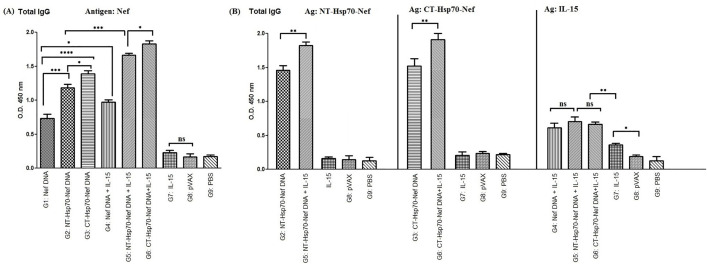
Quantification of total IgG levels elicited by different DNA immunization regimens: A) Antibody response against the Nef antigen; B) IgG response against recombinant NT-Hsp70-Nef, CT-Hsp70-Nef, and IL-15 proteins. Indirect ELISA was performed in duplicate. Data were shown as mean absorbance at 450 nm ± SD. (ns: not significant; *p < 0.05; **p < 0.01; ***p < 0.001; ****p < 0.0001).

Total IgG levels were quantified using ELISA to evaluate the humoral immune response induced by the vaccine formulations. Among the groups tested, the strongest IgG response to the Nef antigen was observed in mice that received the combination of pVAX-CT-Hsp70-Nef and pVAX-IL-15 (G6). The inclusion of Hsp70 domains—either NT or CT—fused to the Nef sequence (G2 and G3) led to significantly enhanced antibody production compared to the group immunized with the Nef construct alone (G1, *p* < 0.001 & *p* < 0.0001). Furthermore, co-delivery of IL-15 *p*lasmid with pVAX-Nef (G4) resulted in a notable increase in IgG levels compared to pVAX-Nef alone (G1, *p* < 0.05; Fig 3A). When IL-15 was co-administered with either NT-Hs*p*70-Nef (G5) or CT-Hsp70-Nef (G6), a further rise in total IgG levels was observed, surpassing that of their counterparts without IL-15 (G2 and G3, respectively; *p* < 0.001). This result underscores the synergistic role of IL-15 in am*p*lifying antibody responses elicited by Hsp70-based constructs. Moreover, enhanced IgG production was more pronounced in mice immunized with CT-Hsp70-Nef (G3 and G6) than in those receiving NT-Hsp70-Nef (G2 and G5; *p* < 0.05), reflecting the su*p*erior adjuvant potential of the CT domain.

To further explore antigen-specific IgG profiles, we measured antibody titers against the purified NT-Hsp70-Nef, CT-Hsp70-Nef, and IL-15 proteins in animals that received these respective antigens. Mice co-immunized with IL-15 and either pVAX-NT-Hsp70-Nef or pVAX-CT-Hsp70-Nef (G5 and G6) exhibited significantly higher IgG responses to the corresponding recombinant proteins than those receiving the constructs without IL-15 (G2 and G3; *p* < 0.01; Fig 3B). No significant differences were observed in IgG recognition of the recombinant IL-15 among groups administered with pVAX-IL-15 in combination with other plasmids (G4, G5, G6; *p* > 0.05). However, these groups displayed increased antibody responses relative to the group that received pVAX-IL-15 alone (G7; *p* < 0.01). Additionally, pVAX-IL-15 alone (G7) triggered a detectable IgG response against the IL-15 antigen that was significantly greater than in the control groups receiving empty vector or PBS (G8 and G9; *p* < 0.05).

### Analysis of cytokine responses

**Fig 4:**
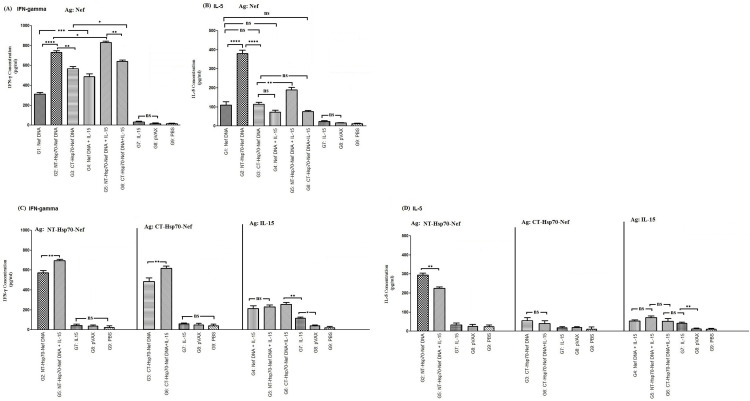
Cytokine profiling of IFN-γ and IL-5 secreted by splenocytes re-stimulated with recombinant proteins: A–B) Re-stimulation with Nef; C–D) Re-stimulation with NT-Hsp70-Nef, CT-Hsp70-Nef, or IL-15. Cytokine levels were measured by sandwich ELISA in duplicate assays per group (ns: non-significant; * *p < *0.05; ** *p < *0.01, *** *p < *0.001, ****p < 0.0001).

To evaluate cellular immune activation following vaccination, levels of IFN-γ and IL-5 were measured in splenocyte cultures after re-stimulation with the Nef antigen. Mice immunized with pVAX-NT-Hsp70-Nef (G2 and G5) exhibited elevated secretion of both cytokines compared to other experimental groups (*p* < 0.05; Fig 4A and B). Importantly, co-administration of IL-15 (G4, G5, and G6) led to increased IFN-γ while concurrently reducing IL-5 levels, suggesting a Th1-skewed immune profile. While groups receiving NT-Hsp70 fusions (G2 and G5) generated more IFN-γ than those immunized with CT-Hsp70-Nef constructs (G3 and G6; *p* < 0.01), the IFN-γ to IL-5 secretion ratio was higher in the CT-Hsp70-Nef immunized groups. This indicates that CT-Hsp70 may more effectively bias responses toward a Th1 phenotype—particularly when paired with IL-15.

Following re-stimulation with the respective recombinant proteins (NT-Hsp70-Nef or CT-Hsp70-Nef), significantly greater IFN-γ production was observed in mice receiving IL-15 co-delivery (G5 and G6) compared to their counterparts lacking IL-15 (G2 and G3; *p* < 0.01). In contrast, no significant differences in IFN-γ secretion were found among IL-15 DNA recipients (G4, G5, G6) following re-stimulation with the recombinant IL-15 itself. Nonetheless, these co-immunized groups (G4, G5, G6) demonstrated enhanced IFN-γ levels compared to mice that received the IL-15 plasmid alone (G7; *p* < 0.01). Additionally, G7 still produced substantially more IFN-γ relative to the baseline controls (G8 and G9; Fig 4C).

Further examination showed that IL-5 levels were significantly reduced in G5 (NT-Hsp70-Nef + IL-15) compared to G2 (NT-Hsp70-Nef alone) when re-stimulated with the NT-Hsp70-Nef protein (*p* < 0.01). However, IL-5 secretion in G6 (CT-Hsp70-Nef + IL-15) was not significantly different from G3 (CT-Hsp70-Nef alone) under the CT-Hsp70-Nef re-stimulation (*p* > 0.05). Importantly, both CT-Hsp70 groups (G3 and G6) showed markedly lower IL-5 levels relative to their NT-Hsp70 counterparts (G2 and G5; *p* < 0.0001), emphasizing the role of CT-Hsp70 in dampening Th2-associated cytokines. Moreover, re-stimulating splenocytes from IL-15-administered groups (G4–G7) with the recombinant IL-15 protein did not lead to significant differences in IL-5 production (*p* > 0.05). Nevertheless, these groups produced considerably higher IL-5 than the control groups (G8 and G9; *p* < 0.01), confirming the bioactivity of the IL-15 plasmid (Fig 4D). As a final observation, co-immunization with IL-15 and antigen-encoding plasmids (G4, G5, G6) led to substantially greater IFN-γ responses compared to IL-15 alone (G7, *p* < 0.01), while G7 maintained significantly higher IFN-γ levels than controls (G8 and G9, *p *< 0.05).

### Assessment of granzyme B production

**Fig 5:**
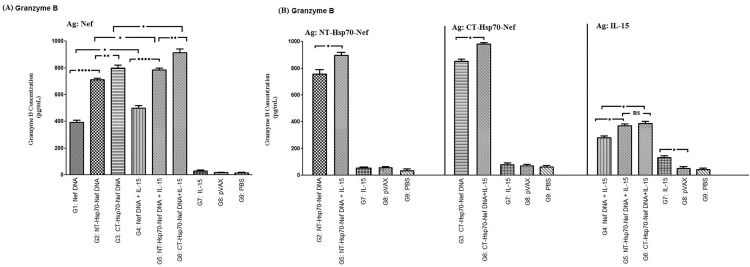
Assessment of granzyme B release by antigen-restimulated effector splenocytes: A) Re-stimulated with the Nef antigen; B) Re-stimulated with NT-Hsp70-Nef, CT-Hsp70-Nef, or IL-15 protein. Data represent sandwich ELISA results performed in triplicate per sample (ns: non-significant; * *p < *0.05; ** *p < *0.01, *** *p < *0.001, ****p < 0.0001).

Granzyme B levels were measured to evaluate CTL-mediated cytotoxic activity following antigen re-stimulation. Groups that received IL-15 in combination with other DNA constructs (G4, G5, and G6) showed significantly increased granzyme B secretion upon Nef protein re-stimulation compared to those immunized with the same constructs lacking IL-15 (G1, G2, and G3; *p* < 0.05). In particular, mice receiving pVAX-CT-Hsp70-Nef with or without IL-15 (G6 and G3) produced markedly higher granzyme B levels than those administered pVAX-NT-Hsp70-Nef equivalents (G5 and G2; *p* < 0.01). Additionally, fusion of either Hsp70 fragment (NT or CT) to Nef significantly enhanced cytolytic potential, as evidenced by increased granzyme B release in groups G2, G3, G5, and G6 compared to groups vaccinated with pVAX-Nef alone or alongside IL-15 (G1 and G4; *p* < 0.0001; Fig 5A).

Upon re-stimulation with recombinant NT-Hsp70-Nef or CT-Hsp70-Nef proteins, the co-immunized groups (G5 and G6) again demonstrated elevated granzyme B secretion compared to their counterparts without IL-15 (G2 and G3; *p* < 0.05; Fig 5B). However, granzyme B secretion did not differ significantly when those same groups were re-stimulated with the recombinant IL-15 protein (*p* > 0.05). Nevertheless, both groups G5 and G6 produced more granzyme B than the group immunized with pVAX-Nef plus IL-15 (G4) under the same IL-15 re-stimulation condition (*p* < 0.05). Moreover, the IL-15-alone group (G7) displayed significantly enhanced granzyme B release relative to the negative control groups (G8 and G9; *p* < 0.05), confirming its immunostimulatory potential.

### *In vitro* analysis of SCR HIV-1-specific cytokine responses

**Fig 6:**
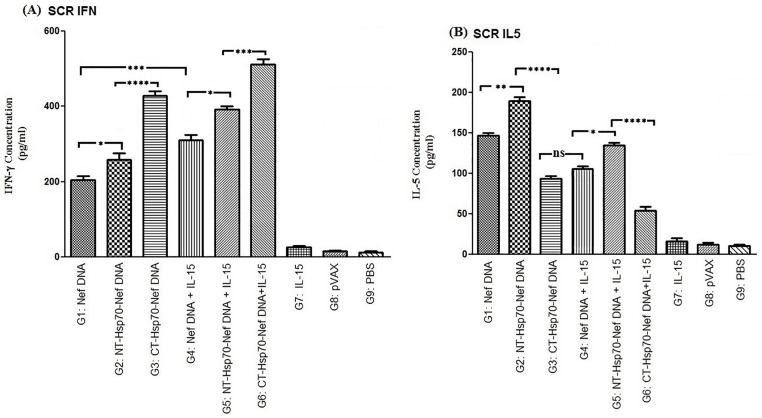
Cytokine secretion from splenocytes exposed to SCR HIV-1. **A)** IFN-γ and **B)** IL-5 levels were compared across vaccinated and control groups. Measurements were performed in duplicate per sample (ns: non-significant; * *p < *0.05; ** *p < *0.01, *** *p < *0.001, ****p < 0.0001).

Following infection of splenocytes with SCR HIV-1, cytokine levels in culture supernatants were measured to assess antigen-specific cellular immune responses. All vaccinated groups exhibited significantly higher IFN-γ and IL-5 secretion compared to control groups (G7, G8, G9; *p* < 0.0001; Fig 6). The highest IFN-γ production was observed in mice immunized with the combined regimen of pVAX-CT-Hsp70-Nef and pVAX-IL-15 (G6). Fusion of Hsp70 domains (CT or NT) to the Nef DNA significantly enhanced IFN-γ levels, regardless of IL-15 inclusion, as compared to the Nef DNA alone (*p* < 0.05). Furthermore, groups receiving IL-15 along with antigenic constructs (G4, G5, G6) showed notably elevated IFN-γ production versus those lacking IL-15 (G1, G2, G3; *p* < 0.0001), indicating synergistic effects from IL-15 co-delivery.

Among the Hsp70 variants, CT-Hsp70 fusion yielded stronger stimulation of IFN-γ compared to NT-Hsp70 when evaluated in infected splenocyte cultures, both in the presence and absence of IL-15 (G3, G6 *vs.* G2, G5; *p* < 0.0001; Fig 6A). In contrast, IL-5 levels were significantly lower in mice vaccinated with CT-Hsp70-Nef, either with (G6) or without (G3) IL-15, suggesting suppression of Th2 responses in these animals. In comparison, the NT-Hsp70-based groups (G2 and G5) displayed elevated IL-5 secretion, highlighting a less favorable Th1/Th2 balance. Moreover, inclusion of IL-15 in the vaccination strategy (G4, G5, G6) reduced IL-5 expression compared to corresponding groups lacking IL-15 (G1, G2, G3), supporting IL-15’s role in downregulating Th2-associated cytokines (Fig 6B).

## Discussion

In the present investigation, we assessed the individual and combined impacts of Hsp70 fragments and the IL-15 cytokine on enhancing antigen-specific immunity and responses against SCR HIV-1, with the aim of advancing an HIV-1 DNA vaccine candidate. DNA vaccines represent a promising approach for eliciting antigen-specific immunity, prompting researchers to concentrate on augmenting their immunogenicity through diverse adjuvants and delivery systems [24]. The IL-15 cytokine has garnered substantial attention as an adjuvant across various preclinical and clinical investigations [6]. Several studies have supported the role of IL-15 as an adjuvant in prophylactic HIV vaccines [25]. This cytokine enhances T cell proliferation, functionality, differentiation, and longevity [26–28]. For instance, Saikh *et al*. demonstrated that administering IL-15 alongside a recombinant staphylococcal enterotoxin B (SEB) vaccine (STEBVax) in mice resulted in a notable, dose-related elevation in antigen-specific antibodies [6]. Similarly, Valkenburga *et al*. developed an innovative vaccine employing live vaccinia virus as a vector to express five H5N1 proteins (namely, HA, NA, M1, M2, and NP) in conjunction with IL-15 as an adjuvant. Their results underscored the effectiveness of this multivalent formulation (Wyeth/IL-15/5Flu) as a broad-spectrum influenza vaccine, generating protective immunity irrespective of neutralizing antibody involvement [29]. Likewise, co-delivery of lentiDNA vaccines incorporating IL-7 or IL-15 genes (CAL-SHIV-IN-IRES IL-7 and CAL-SHIV-IN-IRES IL-15, created by integrating IL-7 or IL-15 into the CAL-SHIV-IN plasmid) markedly amplified vaccine-specific CD4^+^ and CD8^+^ T cell responses in both mice and rhesus macaques, while also boosting antibody-dependent cell-mediated cytotoxicity (ADCC) in plasma and mucosal sites of macaques, outperforming a live-attenuated virus-derived CAL-SHIV-IN lentiDNA vaccine [30]. Along similar lines, a DNA vaccine incorporating HIV-1 *Gag* fused with IL-7 or IL-15 (DermaVir) substantially amplified Gag-specific central and effector memory CD8^+^ T cells in murine models [11,12]. Additionally, co-administration of a vif-deficient simian immunodeficiency virus (SIV) mac239 proviral DNA vaccine (SIV/CMVΔvif) with an optimized rhesus IL-15 (rIL-15) plasmid significantly heightened IFN-γ production and multifunctional CD8^+^ T cell activity in macaques [25]. Separately, subcutaneous delivery of cationic liposomes containing an IL-15-encoding plasmid as an adjuvant, paired with an autologous whole-cell tumor vaccine, achieved substantial tumor growth suppression in mice without adverse effects. These observations indicated that the IL-15 gene markedly strengthened both cell-mediated and antibody-based immunity elicited by autologous whole-cell tumor vaccines in a murine lung cancer model [9]. Furthermore, co-delivery of an IL-15-expressing plasmid (pIL-15) enhanced sustained T cell viability, memory T cell proliferation, and enduring protection from a genetic vaccine based on a plasmid encoding the *Trypanosoma cruzi* trans-sialidase gene (pTS) against fatal *T. cruzi* infection [31].

Our findings demonstrated that co-administration of the HIV-1 Nef DNA construct with the IL-15 DNA construct substantially elevated cellular immune responses, including IFN-γ and granzyme B production, relative to the HIV-1 Nef DNA construct alone. Consistent with this, Kutzler *et al*. reported that vaccination with an optimized IL-15 DNA construct paired with an HIV-1 *gag* DNA construct substantially boosted antigen-specific CD8^+^ T cell expansion and IFN-γ release, along with robust generation of durable CD8^+^ T cell activity [24]. We also observed that mice immunized with pVAX-Nef + pVAX-IL-15 sustained T cell memory function through elevated IFN-γ production compared to those given pVAX-Nef alone. Another investigation found that subcutaneous delivery of SARS-CoV-2 S1 protein alongside IL-15 and Toll-like receptor agonists (MALP-2, poly I: C, and CpG) triggered antibody production against S1 in mice [32]. In our experiments, the co-administration of pVAX-Nef with pVAX-IL-15 resulted in significantly higher total IgG levels compared to pVAX-Nef alone, highlighting the critical role of IL-15 in enhancing Nef-specific humoral immune responses. It was reported that HIV-1-specific IgG antibody responses may contribute to the control of HIV-1 infection [33]. Similarly, Hu *et al*. demonstrated that co-delivery of a composite DNA vaccine with an IL-15-expressing plasmid significantly enhanced cell-mediated immune responses—characterized by increased IgG2a and IFN-γ levels, as well as improved CTL activity—against *Brucella abortus* in a murine model [34]. Furthermore, our results indicated that pVAX-Nef + pVAX-IL-15 promoted IFN-γ and granzyme B release relative to pVAX-Nef, suggesting a stronger bias toward Th1 and CTL-mediated immunity.

Beyond employing IL-15 as a vaccine adjuvant, it has also been applied independently to activate immune responses. For example, co-expression of IL-15/IL-15Rα and CD80 in syngeneic acute myeloid leukemia (AML) vaccines generated potent, durable anti-leukemic immunity, yielding 50% overall survival in murine models [35]. Moreover, immunization with a structurally altered version of human IL-15 (mhIL-15) generated neutralizing antibodies against endogenous IL-15 in non-human primates. Peak neutralizing activity occurred in macaques vaccinated with alum-adjuvanted mhIL-15. Such a strategy shows potential for managing disorders associated with IL-15 overexpression in their pathology [36]. Collectively, published evidence suggests that IL-15 can enhance the initial activation of CD8^⁺^T cells in response to vaccination [8].

Beyond cytokines, HSPs—particularly their truncated forms—have been recognized as powerful adjuvants. For instance, a DNA fragment encoding the *lcrV* gene from *Y. pestis* fused to the domain II of *M. tuberculosis* Hsp70 was cloned, and expressed in *E. coli*, yielding a purified recombinant LcrV-Hsp70 fusion protein via affinity chromatography. This fusion elicited robust humoral immunity and complete protection, surpassing responses from LcrV, Hsp70, or control treatments alone [37]. Similarly, a new recombinant Hsp70-AD fusion demonstrated strong cell-mediated protection against foot-and-mouth disease virus (FMDV) in both mice and swine [38]. Jiang *et al*. reported that linking Hsp70 to the tumor antigen Mage-a1 markedly improved protein vaccine performance, including heightened antigen-specific antibodies, IFN-γ release, CTL function, and antitumor protection against Mage-a1-positive tumors, outperforming Mage-a1 alone or mixed with Hsp70 in mice [17]. Evidence confirms that HSP immunostimulatory properties are concentrated in their N- or C-terminal domains. These compact segments, termed mini-chaperones, can attach to peptides and be internalized via APC receptors such as CD91 and scavenger receptor class A, positioning them as valuable adjuvants for cancer immunotherapy and vaccines by boosting CTL responses to pathogens [19]. As an illustration, Liu *et al*. found that fusing the N-terminal portion of calreticulin (NT-CRT) or the C-terminal segment of Hsp70 (CT-Hsp70) to HPV16 E7 generated robust antigen-specific CTL activity in mice. Moreover, the NT-CRT/E7/CT-Hsp70 fusion promoted strong antitumor effects and anti-angiogenic activity [39]. In another case, the C-terminal region of mycobacterial Hsp70 (residues 359−610) fused to a chronic hepatitis B virus (HBV) antigen in a DNA vaccine substantially enhanced HBsAg-specific Th1 and CTL activity, facilitating clearance of circulating HBsAg and suppression of HBV replication. This Hsp70 domain outperformed full-length Hsp70 as an adjuvant in HBV DNA immunization [40]. Similarly, fusion of the C-terminal fragment of mycobacterial Hsp70 to the malaria antigen EB200 in a DNA vaccine significantly enhanced immune responses in mice [41]. In line with these findings, our study demonstrated that linking the C-terminal domain of human Hsp70 (amino acids 508–641) to the HIV-1 Nef antigen within a DNA vaccine construct markedly increased total IgG levels, IFN-γ (a typical Th1 cytokine)/IL-5 (a typical Th2 cytokine) cytokine ratios, and granzyme B secretion (granzyme B plays a critical role in granule-mediated apoptosis by CTL [42]), compared to either Nef DNA alone or a construct containing the N-terminal segment of Hsp70 (residues 1–387). These results highlight the superior immunostimulatory capacity of the CT-Hsp70 domain in promoting Th1-biased and CTL responses. Comparable outcomes appear in other work; for example, a DNA vaccine fusing CT-mycobacterial Hsp70 to MPT51 (a key secreted *Mycobacterium tuberculosis* protein) triggered greater MPT51-specific IFN-γ release from CD4^+^ T cells and a more robust Th1 response than vaccines with MPT51 alone or MPT51 fused to NT-Hsp70 [43].

Our findings demonstrated that pairing the IL-15 DNA construct with the CT-Hsp70-Nef DNA construct substantially elevated total IgG, IFN-γ, and granzyme B production relative to pairing with the NT-Hsp70-Nef DNA construct. Furthermore, IFN-γ production was significantly higher in splenocytes from SCR HIV-1-infected mice that received CT-Hsp70-Nef DNA in combination with IL-15 DNA, compared to those immunized with NT-Hsp70-Nef DNA plus IL-15 DNA, indicating enhanced and sustained T cell functionality in response to viral challenge. Conversely, IL-5 production was reduced in the group receiving CT-Hsp70-Nef DNA + IL-15 DNA relative to the NT-Hsp70-Nef DNA + IL-15 DNA group following viral exposure, mirroring patterns seen with *in vitro* antigen re-stimulation and pointing to a predominant Th1 profile. Prior work from our lab indicated that CT-Hsp70 provided enhanced adjuvant effects for generating Nef-specific immunity via dendritic cell-based approaches [44]. Additionally, switching the DNA backbone (from pcDNA to pVAX-1, which is suitable for human applications) and omitting a delivery agent (like Rev cell-penetrating peptide) preserved the overall immune response orientation [21,22].

Nef protein was considered as an attractive target in therapeutic HIV vaccine development [21,22,44]. In this context, fusion of either NT-Hsp70 or CT-Hsp70 to the Nef antigen significantly enhanced cellular immune responses compared to antigen alone in DNA-based immunization, emphasizing their role as functional adjuvants. This enhancement was further pronounced in groups receiving Hsp70-Nef fusion constructs in combination with IL-15, which elicited stronger immune responses than the group immunized with Nef and IL-15 co-delivered without Hsp70 fusion. Nevertheless, groups given Nef + IL-15, CT-Hsp70-Nef + IL-15, and NT-Hsp70-Nef + IL-15 DNA constructs elicited significantly stronger cellular immune responses than those receiving Nef, CT-Hsp70-Nef, and NT-Hsp70-Nef DNA constructs, respectively, emphasizing the central contribution of the IL-15 cytokine as a DNA adjuvant. Our findings demonstrated that the highest levels of cellular immune activation and antibody production were observed in the group immunized with the CT-Hsp70-Nef construct in combination with IL-15 DNA, reflecting the synergistic effect of CT-Hsp70 and IL-15 in promoting Th1-biased and CTL responses in mice. Furthermore, this approach sustained protective immunity in splenocytes infected with SCR HIV-1.

In conclusion, our findings highlight the effectiveness of concurrently employing two adjuvants—CT-Hsp70 and IL-15—for augmenting antigen-specific immunity. Such a strategy holds promise as a vaccine candidate for enhancing robust immune defenses against HIV-1 infection.

## Supporting information

S1 File Raw and original Figs 1 and 2. (RAR)
